# Increase in activity of Na^+^, K^+^-ATPase by Porphyrin compounds as treatment for Dysnatremias caused by Diabetes Mellitus

**DOI:** 10.12669/pjms.325.11530

**Published:** 2016

**Authors:** Abdul Hai, Nadeem Kizilbash

**Affiliations:** 1 Abdul Hai, Department of Medical Laboratory Technology, Faculty of Applied Medical Sciences, Northern Border University, Arar, Saudi Arabia; 2 Nadeem Kizilbash, Department of Medical Laboratory Technology, Faculty of Applied Medical Sciences, Northern Border University, Arar, Saudi Arabia

**Keywords:** Na^+^, K^+^-ATPase, Porphyrin derivatives, Diabetes Mellitus, Acetylcholinesterase, Dysnatremias, Acetylcholinesterase (AChE), Tetraphenylporphinesulfonate (TPPS), 5,10,15,20-Tetrakis (4-sulfonatophenyl) porphyrinato Iron(III) Chloride (FeTPPS) and, 5,10,15,20-Tetrakis (4-sulfonatophenyl) porphyrinato Iron(III) nitrosyl Chloride (FeNOTPPS)

## Abstract

**Objective:**

The aim of this study was to test the action of Porphyrin compounds, Tetraphenylporphine sulfonate (TPPS), 5,10,15,20-Tetrakis (4-sulfonatophenyl) porphyrinato Iron(III) Chloride (FeTPPS) and 5,10,15,20-Tetrakis (4-sulfonatophenyl) porphyrinato Iron(III) nitrosyl Chloride (FeNOTPPS), on Na+, K+ -ATPase of cell membrane of erythrocytes.

**Methods:**

Enzymatic assays, measuring the amount of inorganic phosphate produced, were used to estimate the activity of Na^+^, K^+^-ATPase.

**Results:**

The results show that Porphyrin compounds exert an insulin-like effect on Na^+^, K^+^-ATPase. They act by increasing the activity of the membrane-bound enzyme.

**Conclusion:**

All the three Porphyrin compounds increased the activity of erythrocyte Na^+^, K^+^-ATPase. The exact mechanism of action of these compounds is not clear.

## INTRODUCTION

The relationship between blood glucose level and the levels of Na^+^ and K^+^ ions is complex and depends on a number of factors.^1^ Diabetes Mellitus causes dysnatremias^2^ via several mechanisms.^3^,^4^ In Diabetes Mellitus, high glucose level increases serum osmolality which results in the loss of water from the cells and results in a reduction in serum Na^+^ions level by dilution. In diabetic ketoacidosis, ketone bodies (β-hydroxybutyrate and acetoacetate) cause urinary electrolyte losses which can cause hypernatremia.^5^

Na^+^, K^+^-ATPase pumps Na^+^ and K^+^ ions across the cell membranes to maintain the trans-membrane gradients of these ions. Na^+^ ions are pre-dominant in the extracellular fluid. Since Na^+^ ions cannot enter the cells, they produce an osmotic pressure that results in the retention of water in the extracellular fluid. The osmolarity of the extracellular fluid is constant and the amount of Na^+^ in the cells determines the volume of the extracellular fluid. The mechanism of action of Na^+^, K^+^-ATPase involves hydrolysis of ATP to produce a phosphorylated E1P-E2P state. It is coupled to the transport of Na^+^ and K^+^ ions across the cell membrane. The phosphorylated enzyme intermediate is then hydrolyzed and an E2-E1 state is produced to complete the cycle.^6^,^7^

Erythrocytes are known to resemble liver cells. They possess specific receptors for insulin.^8^ It is known that insulin deficiency during Diabetes Mellitus causes membrane alterations in erythrocytes.^9^ A decreased activity of erythrocyte membrane Na^+^, K^+^-ATPase has been reported in Diabetics.^10^ This decreased activity plays a major role in the development of cardiomyopathy.^11^ and may also result in metabolic, cardiovascular, ocular, and neural complications.^12^ Some studies have previously shown that Na^+^, K^+^-ATPase activity is decreased in erythrocytes during Diabetes Mellitus.^13^ Diabetic patients show a 30% reduction in erythrocyte Na^+^, K^+^-ATPase activity as compared to controls. The decrease in ATPase activity can result in the development of Diabetic Neuropathy.

Porphyrins are heterocyclic aromatic compounds. The parent porphyrin is called porphin, and substituted porphines are called porphyrins. We have previously evaluated Porphyrins in the context of photodynamic therapy (PDT) since they strongly absorb visible light in the blue region.^14^ In addition, we have also shown that these compounds serve as inhibitors for Acetylcholinesterase enzyme in erythrocytes and nerve tissue.^15^,^16^ This study was conducted to investigate the three Porphyrin derivatives: Tetraphenylporphinesulfonate (TPPS), 5,10,15,20-Tetrakis (4-sulfonatophenyl) porphyrinato Iron(III) Chloride (FeTPPS) and 5,10,15,20-Tetrakis (4-sulfonatophenyl) porphyrinato Iron(III) nitrosyl Chloride (FeNOTPPS), as candidate compounds for increasing the Na^+^, K^+^-ATPase activity in erythrocytes. The use of these compounds can be useful for the prevention of dysnatremias in Diabetes Mellitus.

## METHODS

### Synthesis of Porphyrin compounds

The three Porphyrin compounds, TPPS, FeTPPS and FeNOTPPS, were synthesized by use of established procedures.^17^ Commercially obtained pyrrole, benzaldehyde and acetone were distilled before use. A stock solution (2.5 M) of BF_3_-ether was prepared in CH_2_Cl_2_.^1^H NMR were recorded in CDCl_3_ using a 400 MHz NMR spectrometer. The UV-Vis. spectra were recorded on a UV-Vis. spectrophotometer.

### Collection of blood samples and isolation of erythrocyte membranes

Blood samples were obtained from 15 volunteers. The exclusion criteria were that none of the subjects suffered from arthritis, hypertension or Diabetes Mellitus. The samples were centrifuged at 4°C for 10 min at 100 g. The erythrocyte membranes were isolated by established procedures.^18^

### Determination of protein content

The erythrocyte membranes were assayed for protein content by the method of Lowry et al.^19^

### Determination of Na^+^, K^+^-ATPase activity

The enzymatic activity of Na^+^, K^+^-ATPase was measured using published protocols.^20^ The amount of inorganic phosphate produced was estimated by the method of Fiske and Subbarow.^21^

### In vitro experiments of Porphyrin compounds with Na^+^, K^+^-ATPase

The effect of Porphyrin compounds on Na^+^, K^+^-ATPase activity was investigated using published protocols.^20^ A stock solution (10 mM) of Porphyrin compounds in 5% ethanol was used. This solution was diluted further with water before use. The *in vitro* effect of Porphyrin compounds was monitored by incubating the reaction mixture for 60 min at 37°C.

### Statistical analysis

Statistical analysis was performed by the use of MedCalc® program version 8.1.0.0.^22^ The enzyme activity data was expressed as mean ± standard deviation. *P*-value was considered significant if it was less than 0.05.

### Ethical Considerations

The Vice Dean (Academics), Faculty of Applied Medical Sciences, Northern Border University reviewed the ethical considerations for this project and found them to have been satisfied.

## RESULTS

Treatment of erythrocyte Na^+^, K^+^-ATPase with the three Porphyrin compounds resulted in an increase in activity of the enzyme significantly (Table-I). The observation of increased Na^+^, K^+^-ATPase activity in response to Tetraphenylporphinesulfonate (TPPS), 5,10,15,20-Tetrakis (4-sulfonatophenyl) porphyrinato Iron(III) Chloride (FeTPPS) and 5,10,15,20-Tetrakis (4-sulfonatophenyl) porphyrinato Iron(III) nitrosyl Chloride (FeNOTPPS) pre-treatment is interesting since insulin also causes an increase in Na^+^-K^+^-ATPase activity in erythrocytes of Diabetics. Further work is needed to explain the effect of Porphyrin compounds on erythrocyte Na^+^, K^+^-ATPase and also on other membrane bound enzymes.

**Table-I:** Erythrocyte membrane Na^+^-K^+^-ATPase activity in human erythrocytes before and after in vitro treatment with synthetic compounds: TPPS, FeTPPS and FeTPPSNO.

		*Enzyme Activity	*P-value*

Control Solution		0.010 ± 0.001	0.02

*Compound*	*Concentration (M)*		
TPPS	10^-10^	0.011 ± 0.001	0.002
	10^-9^	0.013 ± 0.003	0.05
	10^-8^	0.018 ± 0.002	0.02
	10^-7^	0.022 ± 0.002	0.03
	10^-6^	0.027 ± 0.003	0.04
	10^-5^	0.033 ± 0.007	0.002
	10^-4^	0.036 ± 0.003	0.02
	10^-3^	0.039 ± 0.003	0.03
FeTPPS	10^-10^	0.011 ± 0.002	0.04
	10^-9^	0.012 ± 0.002	0.002
	10^-8^	0.015 ± 0.002	0.02
	10^-7^	0.018 ± 0.002	0.03
	10^-6^	0.024 ± 0.003	0.002
	10^-5^	0.028 ± 0.005	0.05
	10^-4^	0.032 ± 0.004	0.02
	10^-3^	0.036 ± 0.003	0.03
FeNOTPPS	10^-10^	0.011 ± 0.001	0.05
	10^-9^	0.011 ± 0.001	0.002
	10^-8^	0.014 ± 0.003	0.05
	10^-7^	0.016 ± 0.002	0.02
	10^-6^	0.021 ± 0.003	0.04
	10^-5^	0.025 ± 0.002	0.002
	10^-4^	0.028 ± 0.003	0.02
	10^-3^	0.033 ± 0.004	0.04

* Each value is the mean of at least 3-4 independent experiments. Values are expressed as mean +/- standard deviation. Na^+^, K^+^-ATPase activity was measured as described earlier.^11^

## DISCUSSION

The results show that all the three Porphyrin compounds caused an increase in the activity of erythrocyte Na^+^, K^+^-ATPase (Table-I). The mechanism underlying the non-neuronal effects of Porphyrin compounds is unclear. It is possible that these lipophilic compounds insert into the plasma membrane of erythrocytes and alter their membrane fluidity. It has previously been reported that erythrocytes exhibit different properties in Diabetic patients.^12^,^23^ These cells have decreased deformability,^24^ increased membrane viscosity^25^ and increased erythrocyte aggregation.^26^ The decreased Na^+^,K^+^-ATPase activity observed in the erythrocyte membrane of Diabetics leads to an increase in Na^+^ and Ca^+2^ ions. The decrease in plasma Na^+^ level and decreased activity of erythrocyte membrane Na^+^, K^+^ -ATPase can disturb ion homeostasis of cells. ROS-induced oxidation damages the erythrocyte membrane Na^+^, K^+^-ATPase and results in an increase in the efflux of K^+^ from the cells that results in cell lysis.

The intra-cellular increase in Na^+^ and Ca^+2^ ions that can decrease the deformability of erythrocytes in Diabetics.^12^ The diabetes-induced decrease in Na^+^, K^+^-ATPase activity may also be due to hyperglycemia that increases glycosylation of the β subunit of Na^+^, K^+^-ATPase^27^ or there can be a defect in myo-inositol metabolism that changes the lipid content of the cell membranes.^28^ However, a direct effect of hyperglycemia on this enzyme’s activity has never been proven. In type 1 diabetic patients, erythrocyte membrane Na^+^, K^+^-ATPase activity is not related to the plasma glucose, HbA_1c_, plasma triglyceride, or total cholesterol levels. In type 2 diabetes, this enzyme’s activity is less reduced than in type 1 diabetes despite similar HbA_1c_ values.^29^ A similar observation has been made for Na^+^, K^+^-ATPase activity in the sciatic nerve in type 2 Diabetic BBZDR rats compared to type 1 Diabetic BB rats.^30^

## CONCLUSION

The three Porphyrin compounds, TPPS, FeTPPS and FeNOTPPS, increased the activity of erythrocyte Na^+^, K^+^-ATPase. Although, Porphyrin compounds seem to mimic insulin in their effect on Na^+^, K^+^-ATPase, it is difficult to predict the mechanism of action. It is possible that these lipophilic compounds bind to the plasma membrane of erythrocytes and change the membrane fluidity. The findings being reported here prove the importance of the non-neuronal effects of Porphyrin compounds.
